# Early Success is not a Prerequisite for Success at the Adult Age in Spanish Sprinters

**DOI:** 10.5114/jhk/168284

**Published:** 2023-07-06

**Authors:** Aaron Agudo-Ortega, Jose Maria Gonzalez-Rave, Juan Jose Salinero

**Affiliations:** 1 Sports Training Laboratory, Faculty of Sports Sciences, University of Castilla La Mancha, Toledo, Spain.

**Keywords:** sport performance, athletics, transition rate, early specialization, success prediction

## Abstract

The aim of the study was to describe the relationship between success in junior and senior categories in sprint events. An observational and longitudinal analysis was carried out using rankings of the Royal Spanish Athletics Federation database. We analysed 547 sprinters (238 women and 309 men) from their U14 to senior stage who ranked in top-20 on at least one occasion during the period 2004 to 2021. The first entry in top-20 occurred mostly in U14 (44.4%, 243), and the frequency was progressively lower: 26.7% (146) in U16; 20.8% (114) in U18; 5.1% (28) in U20; 2% (11) in U23; and 0.9% (5) in the senior category. A similar tendency was observed in male and female athletes. Only 3.8% (9) of top-20 U14 athletes reached the senior elite stage, increasing this percentage in subsequent categories: 7.4% (15) in U16; 10.6% (24) in U18; 20.9% (32) in U20, and 31.4% (32) from U23 to the senior category. Data from female athletes showed higher maintenance of top-20 status from early categories to senior age. We conclude that even though the first entry into the national top-20 in sprint events occurs early in most cases, success in these initial stages is not a prerequisite for reaching top-20 positions in the senior category.

## Introduction

The athletes´ pathway to elite performance can involve years of deliberate practice (Baker and Young, 2014), and it is influenced by numerous internal and external factors (Suppiah et al., 2015; Till and Baker, 2020). Furthermore, the identification and selection of elite athletes occur during their developmental years (Cuba-Dorado et al., 2020; Johnston et al., 2021; Till and Baker, 2020). Different models for sporting development have been suggested: early specialization, late investment and playful training, a late entry into the main sport, and sampling in playful training (Storm et al., 2012). Although there are troubling inconsistencies in the definitions of early specialization, some key points refer to the choice of the main sport (before age 12), and single sport participation (Mosher et al., 2020), based on the assumption that performance in younger categories may be a good predictor of an athlete's potential and future expert performance (Jayanthi et al., 2019), basing its validity on the models of accumulation of training hours, i.e., deliberate practice (Ericsson, 2008; Macnamara et al., 2014; Thomas et al., 2019). Late specialization is understood as specialization in a single sport and intensification of specialized practice directed at older ages (Güllich et al., 2022). The International Olympic Committee (IOC) in its 2015 declaration promoted late specialization to achieve the full development of the capabilities of athletes (Bergeron et al., 2015). In agreement with the IOC declaration, several studies have shown how early specialization does not generate advantages over late specialization (Feeley et al., 2016; Kliethermes et al., 2020; Siembida et al., 2021; Vaeyens et al., 2009; van Rens et al., 2015).

A correct selection model provides us with the necessary information to establish realistic developmental goals and expectations according to the needs of athletes (Güllich, 2017; Tønnessen et al., 2015). Some authors have found that an earlier onset and a higher volume of discipline-specific training and competition during adolescence do not need necessarily to be associated with greater success in senior international elite sports (Barth et al., 2022; Boccia et al., 2017, 2019; Kliethermes et al., 2020; Moesch et al., 2011). Considering this last statement, those authors have shown a start in the sport at an older age of athletes who achieved great success in the senior category, which entailed a greater sports background in other sports and presented slower initial progress compared to their counterparts at the national level. In sprinters, Boccia et al. (2019) found that entering sport-specific competitions later and lengthening the sports career beyond the age of 23–25 years may be an important factor to achieve the top-level performance in sprinters. National (Boccia et al., 2019) and international-class top-level athletes (Boccia et al., 2020) achieved their personal best around this age range, and this occurred earlier than in their counterparts of a lower performance level.

Nowadays, small performance transfer between junior and senior categories has been observed in team sports such as rugby, football and volleyball (Barreiros et al., 2014; Durandt et al., 2011), and in individual sports such as swimming, cycling, judo, taekwondo, boxing and wrestling (Barreiros et al., 2014; Brustio et al., 2021; Cesanelli et al., 2021; Li et al., 2018; Yustres et al., 2021).

In the same way, in athletics, this transition rate is scarce. In middle- and long-distance runners, only 39% of the finalist athletes in the world junior world championship in 2002 (World Athletics U20 or WJC) maintained the elite status in subsequent years as senior athletes (Pizzuto et al., 2017). Bezuglov et al. (2022) analysed all athletics events and found that from 17,435 U18 athletes ranked in world top-100, only 23.5% entered the top-100 as senior athletes. Boccia et al. (2021) analysed the transition rate for top-50 world jumpers from U18 to the senior category, where only 8% of male and 16% of female top-50 ranked at the age of 16 maintained this status as senior athletes. Boccia et al. (2020) analysed U18 top-50 world sprinters, and only one out of five (17% of male and 21% of female) managed to be included among top-50 when they reached their senior stage. Therefore, only a minority of youth successful athletes maintain their status at the senior category (Bezuglov et al., 2022; Boccia et al., 2020, 2021).

At a national level, Boccia et al. (2017, 2019) analysed the career of Italian athletes, providing that less than 25% of elite U14 athletes (except women—long jumpers, where it was 38%) maintained their status in the senior category. In the United Kingdom, a minority of 100 m sprinters (one out of ten in U15 males and one out of four in U15 females) retained their top-20 status even in U20 (Kearney and Hayes, 2018).

In summary, the existing research shows to a greater or lesser extent that early-stage success is not a prerequisite for future success as an elite athlete and, therefore, the transition rate from junior to senior is low. However, most of the current research in this field has analysed the transition rate only from U18 to the senior category (Bezuglov et al., 2022; Boccia et al., 2020, 2021; Pizzuto et al., 2017), or from U13 to U20 (Kearney and Hayes, 2018). Only few studies have analysed this process from U14 to the senior category (Boccia et al., 2017, 2019), but none of them examined the transition from U14 to the senior category including the U23 transition.

Bearing this in mind, this study aimed to quantify the transition rate of success from junior (from U14 to U23) to senior categories in sprint events in Spain.

## Methods

### Participants

We analysed 547 sprint athletes (238 women and 309 men) ranked in top-20 at any grade on at least one occasion during the period 2004 to 2021.

### Design and Procedures

An observational and longitudinal analysis was conducted to investigate the relationship between junior and senior success (i.e., transition rate).

The data were acquired from the public database of the Royal Spanish Athletics Federation (RFEA), https://www.rfea.es/web/estadisticas/ranking.asp, (access date: 07 February 2022) collecting the best personal times of the different athletics competitions from the U14 to the senior category in Spain since the 2004/2005 season divided by sex, type (indoor or outdoor events), season, category and specific event, including both manual and electronic times and both legal and illegal wind. The athlete's name, ranking, mark, date of birth, and category, among other data of all athletes that competing in Spain each season, were collected. Due to the open-data source employed for data collection, the local Ethics Committee approval was not required.

All data from the 2004/2005 season to 2021 for all categories was downloaded in Microsoft Excel spreadsheets for 100 m, 200 m and 400 m athletes (and 80 m, 150 m and 300 m in junior categories). We removed from the database those results obtained with illegal wind (+2 m/s) and athletes of other nationalities, and selected athletes that entered at any time the top-20 at any stage. We selected athletes born between 1993 and 1996 because we could collect rankings for these athletes from their first year in U14 until they remained at least for three years as senior athletes (23 to 25 years), leaving a final sample of 547 athletes. Previous studies have identified this age range as the age of peak performance for sprint athletes at the national level (Boccia et al., 2019). Each athlete was only counted once per age category. The ranking identified for each athlete at each category was the best achieved across the two (or three in U23) years within this category, and was categorized as top-20 (ranked 1 to 20), “21 to 50” or “Not ranked” (out of top-50). This methodology was used in previous similar studies in Italy and the United Kingdom (Boccia et al., 2017, 2021; Kearney and Hayes, 2018).

### Statistical Analysis

Data are presented as relative and absolute frequencies (%, n). To analyse the frequency in each category, and the transition rate in subsequent categories: “top-20”; “21 to 50” or “Not ranked”, contingency tables were used. All calculations were performed with SPSS 28.0.

## Results

**Figure 1:**
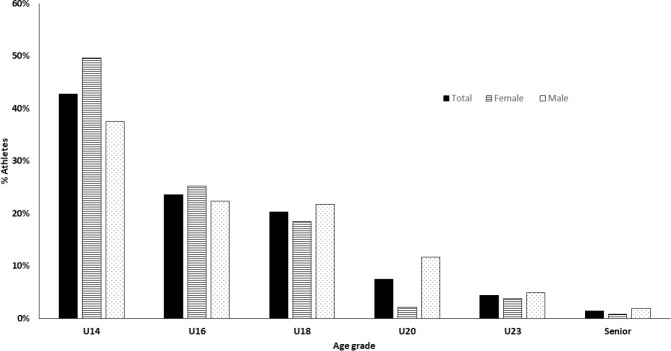
First entry category in top-20.

The first entry in top-20 occurred mostly in U14 (42.8%, 234) (Figure 1), and the frequency of athletes appearing for the first time in top-20 in the following categories was progressively lower: 23.6% (129) in U16, 20.2% (111) in U18, 7.5% (41) in U20, 4.4% (24) in U23, and 1.4% (8) in the senior category. A similar tendency was observed in male and female athletes, although a higher proportion of female athletes entered first at the early age, U14 (49.6% female vs. 37.5% male).

**Figure 2:**
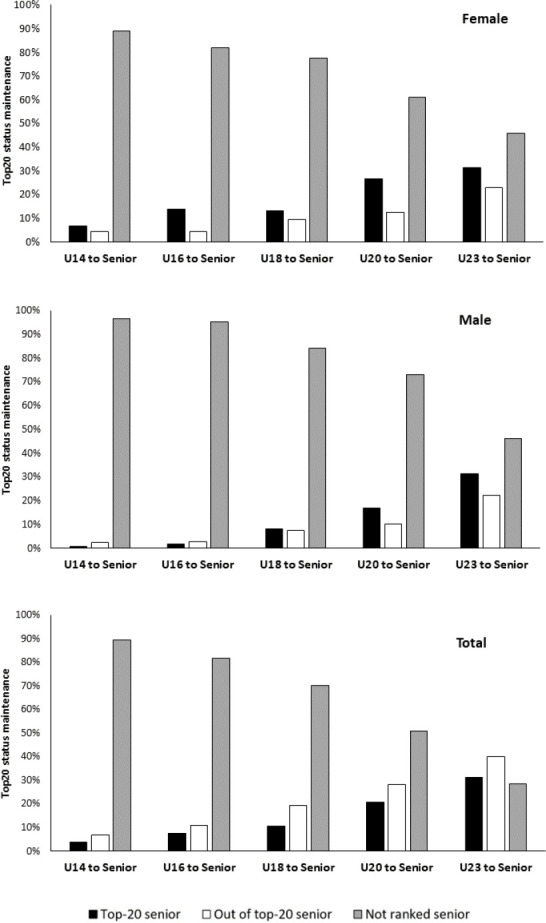
Proportion of top-20 ranked athletes at junior categories who retained the top-20 in the senior ranking.

Figure 2 shows the proportion of athletes who, ranked in top-20 in any of the different junior categories, achieved the top-20 position in the senior category from all (lower panel), male (middle) and female athletes (upper panel). Overall, the percentage of top-20 athletes who maintained this status increased as we approached the senior category: 3.8% (9) in U14, 7.4% (15) in U16, 10.6% (24) in U18, 20.9% (32) in U20, and 31.4% (32) from U23 to the senior category. Data from female athletes showed a higher maintenance of top-20 status from early categories to senior age, except for U23 to the senior category where the figures were almost identical (31.3% in females vs. 31.5% in males).

A secondary analysis of the progression from junior categories with their subsequent categories is shown in Table 1. The first row shows the progression of athletes who, ranked in the top-20 in U14, managed to progress whilst maintaining this level in subsequent categories. Of the total number of top-20 athletes in U14 (234), 31.2% (73) were in the top-20 in U16, 24.4% (57) in U18, 15.4% (36) in U20, and 9% (21) in U23. In U16 (second row) of the total number of top-20 athletes (202), 53.5% (108) remained in the top-20 in U18, 27.5% (55) in U20 and 16.3% (33) in U23. The same pattern occurred in U18, as of the total number of top-20 athletes in this category (228), 45.6% (104) remained in the top-20 in U20 and 26.8% (61) in U23. In U20 of the total number of athletes who were ranked in the top-20 in this category (153), almost one out of every two lost the top-20 spot in U23 (43.1%, 66). Again, females showed higher transition rates between subsequent categories than males (the only exception was from U14 to U16).

**Table 1:** Proportion of top-20 status maintenance between junior categories with their subsequent categories.

		FEMALE				MALE					TOTAL			
		Top-20	21 to 50	Not ranked	Total	Top-20	21 to 50	Not ranked	Total		Top-20	21 to 50	Not ranked	Total
**U14 top-20**	**to U16**	35	19	64	118	38	17	61		116	73	36	125	234
		29.7%	16.1%	54.2%	100%	32.8%	14.7%	52.6%		100%	31.2%	15.4%	53.4%	100%
	**to U18**	31	13	74	118	26	13	77		116	57	26	151	234
		26.3%	11.0%	62.7%	100%	22.4%	11.2%	66.4%		100%	24.4%	11.1%	64.5%	100%
	**to U20**	20	10	88	118	16	6	94		116	36	16	182	234
		16.9%	8.5%	74.6%	100%	13.8%	5.2%	81.0%		100%	15.4%	6.8%	77.8%	100%
	**to U23**	14	7	97	118	7	2	107		116	21	9	204	234
		11.9%	5.9%	82.2%	100%	6.0%	1.7%	92.2%		100%	9%	3.9%	87.2%	100%
**U16 top-20**	**to U18**	59	23	13	95	49	28	30		107	108	51	43	202
		62.1%	24.2%	13.7%	100%	45.8%	26.2%	28.0%		100%	53.5%	25.3%	21.3%	100%
	**to U20**	30	16	49	95	25	16	66		107	55	32	115	202
		31.6%	16.8%	51.6%	100%	23.4%	15.0%	61.7%		100%	27.2%	15.8%	56.9%	100%
	**to U23**	24	8	63	95	9	9	89		107	33	17	152	202
		25.3%	8.4%	66.3%	100%	8.4%	8.4%	83.2%		100%	16.3%	8.4%	75.3%	100%
**U18 top-20**	**to U20**	55	20	32	107	49	32	40		121	104	52	72	228
		51.4%	18.7%	29.9%	100%	40.5%	26.4%	33.1%		100%	45.6%	22.8%	31.6%	100%
	**to U23**	35	18	54	107	26	15	80		121	61	33	134	228
		32.7%	16.8%	50.5%	100%	21.5%	12.4%	66.1%		100%	26.8%	14.5%	58.8%	100%
**U20 top-20**	**to U23**	32	14	18	64	34	22	33		89	66	36	51	153
		50.0%	21.9%	28.1%	100%	38.2%	24.7%	37.1%		100%	43.1%	23.5%	33.3%	100%

## Discussion

The aim of this study was to analyse the transition rate from junior to senior categories. Data analysis from 547 Spanish sprint athletes showed that: 1) first entry in the top-20 position occurred mainly at early ages (Figure 1); 2) the hardest steps to overcome were from U14 to U16, and from U23 to the senior category (Figure 2 and Table 1); and 3) success in junior categories was not a good predictor of success in the senior category (i.e., low transition rate, Figure 2).

The first entry into the top-20 occurred mainly between 12–13 years of age (42.8%), and only one out of three (33.6%) did so after 15 years of age. We understand that at junior ages, training specialization is not one of the principal factors of high performance. At this age, entering the top-20 status is influenced by growth, maturation, development (Malina, 2014) and relative age effect (Brazo-Sayavera et al., 2017; Kearney et al., 2018). Perhaps some of the top-20 athletes in U14 that were conditioned by those factors came out of this privileged position after this advantage had been minimised because girls achieved a plateau between 13 and 15 and boys between 16 and 18 years old (Dobosz et al., 2015; Malina et al., 2010; Tønnessen et al., 2015). Although most athletes reach the top-20 very early, the transition from U14 to U16 is a difficult challenge. In fact, only 31.2% of athletes ranked in top-20 at U14 maintained this status in the following category, while transition rates in the subsequent stages were higher, ranging from 43.1% (U20 to U23) to 53.5% (U16 to U18). In the United Kingdom, Kearney and Hayes (2018) showed similar transition rates from U13 to U15 in the 100 m event (26.0% and 33.1% for males and females vs. 32.8% and 29.7% for males and females in our study from U14 to U16, respectively). However, they found lower values from U15 to U17 (31.8% and 36.8% for male and female athletes, respectively) in contrast to 45.8% and 62.1% from U16 to U18 found in Spanish sprinters, although we should consider the different cut-off points for the age grades between UK and Spain. These results could be explained by the potential advantage of early biological maturation (Malina, 2014) and the relative age effect (Brazo-Sayavera et al., 2017; Kearney et al., 2018), as we have discussed above, understanding that these athletes are more likely to be successful early and not later (McCarthy and Collins, 2014; McCarthy et al., 2022).

Again, the transition rate from U23 to the senior category reached only 31.4%, being the other major wall in the sporting career. To the authors’ knowledge, no studies so far have analysed transition rates from U23 to the senior category. Previous research in athletics analysed transition rates from U18 or U20 to the senior category (Bezuglov et al., 2022; Boccia et al., 2017, 2021), but did not analyse the specific transition from U23 to the senior category. Our data showed that from the U18 category, access to the senior elite was achieved by only 10.6% of the sample (8.3% males and 13.1% females). This figure is far from that in world elite sprinters, where it was found that 17% of male and 21% of female top-50 athletes, subsequently became elite (top-50) in the senior category (Boccia et al., 2020). Similar data have been found in other international athletics sports disciplines. Bezuglov et al. (2022) found that only 23.5% of top-100 track and field athletes at U18 maintained their status at a senior level. From U20, the transition rate reached 35.4%, thus it would be reasonable to think that transition from U23 would be higher than this percentage in this elite sample. However, our data show lower figures: 20.9% from U20 (16.9% males and 26.6% females) and 31.4% from U23 (31.3% females and 31.5% males). We believe that, considering Spanish athletics, where the social and economic repercussions are more limited, there is a greater dropout rate at this age. This could be because from 18 years of age athletes must combine their sporting life with higher education, work and/or family life, and 22–23 years is the usual age to finish higher-education and start working, especially if sport cannot be a way of life, as is the case for most athletes in Spain. These data reinforce the importance of being cautious about extrapolating these transition rates from the national to the international level, because the international top-50 or top-100 could probably be top-3 or less at a national level rather than the top-20.

Despite the main access to top-20 at early ages, success in junior categories is not a good predictor of success at the senior level. This early success does not correspond to a subsequent conversion into high-performance athletes as our results show that only 3.8% of top-20 athletes in U14 maintained this elite status in the senior category. Similarly, this low conversion rate of sporting success has been observed in other sports, such as swimming (Brustio et al., 2021), rugby (Durandt et al., 2011), football, volleyball and judo (Barreiros et al., 2014). In athletics, in Italian long and high jumpers, it was observed that only 17% maintained their elite status (75^th^ percentile or higher) between U14 and the senior category (Boccia et al., 2017), which are much higher figures than 3.8% (0.9% male and 6.8% female) found in our analysis. This higher transition rate could be due to the high technical component of these events, which leads to greater specialization in these disciplines. A similar study in the UK (Kearney and Hayes, 2018) found that in 100 m events, the percentage of athletes who maintained top-20 status from U13 to U15 was 26% and 33.1%, from U13 to U17 it was 13.0% and 25.0%, and from U13 to U20 it was 3.1% and 21.2%, for males and females, respectively. Those authors did not continue their study up to U23 and the senior category, as we did in our study of Spanish athletes. However, we can speculate that percentages of achieving the senior category, considering the results of the aforementioned study, would be even lower than those found in our research.

Thus, as we have observed in our study, the percentage of junior top-20 athletes who achieve elite positions in the senior category is lower in Spanish sprinters than previous world-class sprinters (Boccia et al., 2020). Therefore, given our results and the available research, it seems clear that success in junior categories is not related to success in the senior category, and there is no basis for early specialization despite the fact that some stakeholders (athletes and parents mainly) could believe that successful senior athletes achieved success in junior categories (Brooks et al., 2018; Kearney et al., 2020). For this reason, training and competition in junior categories should be aimed at consolidating the bases on which to develop specialized training at later stages.

Due to the characteristics of the study design (i.e., observational and cross-sectional), outcomes of this research are limited to some extent. One of the limitations was the time restriction of the data available in the national ranking. Since it is only available from 2004–05, to include the entire sporting career of the athletes, from the first available category (U14) to the senior category, we were forced to restrict the sample to athletes born between 1993 and 1996. Second, when an athlete was not listed, we could not determine whether he had dropped out of the sport, had not competed at a sufficient level, or had moved to another athletic discipline. We only analysed sprint performances, thus changes in the athletic discipline (e.g., change to or from jumping events or longer running events) could have affected our outcomes. However, we assume that the sample size allows us to offer a good framework for studying the progression of sprint athletes in Spain.

## Conclusions

Given the results obtained, we conclude that even though the first entry into the national top-20 in sprint events occurs early in most cases, success in these initial stages is not a prerequisite for reaching privileged positions (top-20) in the senior category, the hardest steps to overcome being from U14 to U16, and from U23 to senior categories.

As a practical implication of this study, we would advise parents and/or coaches to avoid early specialization, remembering that success in junior categories is not related to success in the senior category. It is important for the sport governing bodies to create a solid sport system that provides adherence to practice, developing the foundations for a future specialised learning and training.
